# Comparative Analysis of Environmental and Host-Associated Microbiome in *Odorrana schmackeri* (Auran: Ranidae): Insights into Tissue-Specific Colonization and Microbial Adaptation

**DOI:** 10.3390/microorganisms13122725

**Published:** 2025-11-29

**Authors:** Dongyu Han, Ruinan Zhao, Xinyue Yang, Tonghang Wang, Zike Li, Mengyao Zhu, Qingya Yang, Yanfu Qu, Xiaohong Chen, Zhuo Chen

**Affiliations:** 1 The Observation and Research Field Station of Taihang Mountain Forest Ecosystems of Henan Province, College of Life Sciences, Henan Normal University, Xinxiang 453007, China; handongyu1998@163.com (D.H.); zrn202411@163.com (R.Z.); yangxy08051005@163.com (X.Y.); wang15560233063@163.com (T.W.); zikeli1014@163.com (Z.L.); m17337353579@163.com (M.Z.); yangqy030@163.com (Q.Y.); 2 Herpetological Research Center, College of Life Sciences, Nanjing Normal University, Nanjing 210023, China; quyanfu@njnu.edu.cn

**Keywords:** amphibian, *Odorrana schmackeri*, microbiome, tissue-specific microbial niches, sexual dimorphism

## Abstract

Amphibian microbial communities are known to be shaped by host physiology and environmental factors, yet the relative roles of sexual dimorphism and tissue specialization remain poorly understood. Using 16S rRNA gene sequencing, we compared the gastrointestinal and integumentary microbiomes of a monomorphic Chinese frog population, *Odorrana schmackeri*, inhabiting identical montane streams. Our results showed distinct phylogenetic stratification between niches: Proteobacteria dominated both environmental microbiota and *O. schmackeri* gut and skin microbiotas but with differential sub-phylum specialization. The soil microbiota was dominated by *unclassified_Vicinamibacteraceae*, the water microbiota was *Limnohabitans*-dominated, the skin microbiota was dominated by *Bordetella*, and the gut microbiota was led by *Acinetobacter*. Alpha diversity analysis revealed significant tissue- and environmental-based divergences but no sexual differentiation, a pattern confirmed by beta diversity assessments showing stronger microbial community separation by tissue and environmental compartmentalization than by sex. Functional metagenomic prediction indicated convergent enrichment of metabolic pathways across host-associated and environmental communities. These results suggest that microbial community structure in *O. schmackeri* is principally governed by tissue-specific ecological selection pressures rather than host sexual characteristics. Our findings enhance understanding of microbiome assembly rules in vertebrate ectotherms and identify potential connections between microbiota in different ecological niches.

## 1. Introduction

The metacommunity dynamics of cutaneous and enteric microbiomes have become a central focus in host–microbe symbiosis research across vertebrate lineages. Previous studies on the gut and skin microbiota of mammals [1,2], birds [3,4,5], reptiles [6,7], fish [8,9] and invertebrates [10,11] collectively demonstrate the beneficial role of these microbial communities in promoting animal health. Amphibians, bridging aquatic and terrestrial ecosystems [12,13], provide unique insights into vertebrate ecoevolutionary transitions. Their dual-phase life histories, characterized by niche shifts between aquatic larvae and terrestrial adult [14,15], create dynamic microbial transmission bottlenecks. Their ectothermic physiology and permeable skin make them exceptional biological sentinels for environmental change [16,17,18], with microbiome alterations serving as early-warning biomarkers [19,20,21,22]. Thus, amphibian microbiome research sits at the intersection of evolutionary developmental biology and conservation prioritization [16,17,18], offering frameworks to understand host–microbe–environment interactions.

Recent studies have shown that amphibian gut and skin microbiota play critical roles in host–pathogen interactions, physiological regulation, and environmental adaptation, but there is still significant knowledge gaps compared to other vertebrate groups [23,24,25,26]. Comparative analyses reveal Proteobacteria, Bacteroidetes, Actinobacteria and Firmicutes as the dominant phyla across both gut and skin microbiomes [19,20,27,28,29], with dynamic compositional shifts occurring across developmental stages and environmental gradients [30,31,32]. The gut microbiota functions as an essential metabolic interface, mediating host health through facilitating nutrient acquisition and energy metabolism [33,34], synthesizing vitamins [35], and modulating immune system development [36]. These microbial-host interactions ultimately influence individual fitness and population dynamics [37,38]. Of particular ecological significance, amphibian skin microbiota constitutes a frontline defense system against pathogens. Specific taxa, such as *Arthrobacter* and *Bacillus*, demonstrate anti-fungal activity against *Batrachochytrium dendrobatid* (Bd) (a key driver of global amphibian declines) through both direct inhibition [39] and secretions of Bd-suppressive metabolites [40,41]. Moreover, microbial-derived antimicrobial peptides provide broad-spectrum protection against cutaneous infections [42,43]. Recent evidence also positions skin microbiome profiles as bioindicators of environmental stressors, enabling monitoring of habitat quality and climate change impacts [17,44,45].

The composition and functional dynamics of amphibian gut and skin microbiota are influenced by the synergistic interactions between intrinsic host factors (e.g., developmental stage, health status) [18,46,47] and extrinsic environmental drivers (e.g., diet, habitat quality, captivity effects) [48,49,50]. Ontogenetic shifts drive microbial succession, with skin microbiota complexity increasing substantially during metamorphosis [51], while captivity-induced dietary modifications reduce gut and skin microbial diversity compared to wild counterparts [50,52]. These microbial communities exhibit remarkable environmental sensitivity [53,54,55], serving as bioindicators of host physiology and ecological pressures [18,51,56]. The gut microbiome profiles reflect niche adaptation through pesticide degradation in agricultural species [29] and hibernation-associated energy metabolism [57], whereas skin microbiota predict disease susceptibility through climate-mediated functional shifts, such as ABC transporter fluctuations in response to precipitation and thermal extremes [58,59]. This plasticity underscores their dual role in maintaining host fitness while mirroring ecosystem health under anthropogenic and climatic stressors. However, emerging evidence suggests limited investigation into sex-mediated divergence of amphibian gut and skin microbiota below the class level [60,61,62]. While phylum- and class-level compositions remain conserved between sexes, significant differentiation emerges at finer taxonomic resolutions. In *Odorrana tormota*, operational taxonomic units (OTUs) within the same order exhibit marked sexual dimorphism, despite comparable alpha diversity [62]. This pattern extends to gut microbiota, where intersexual beta diversity in *Pelophylax nigromaculatus* manifests predominantly at genus/species levels rather than higher taxa [60]. Such findings underscore the necessity to characterize host–microbe interactions through sex-specific lenses, particularly given potential hormonal modulation of microbial niches [60,62,63,64].

**Figure 1:**
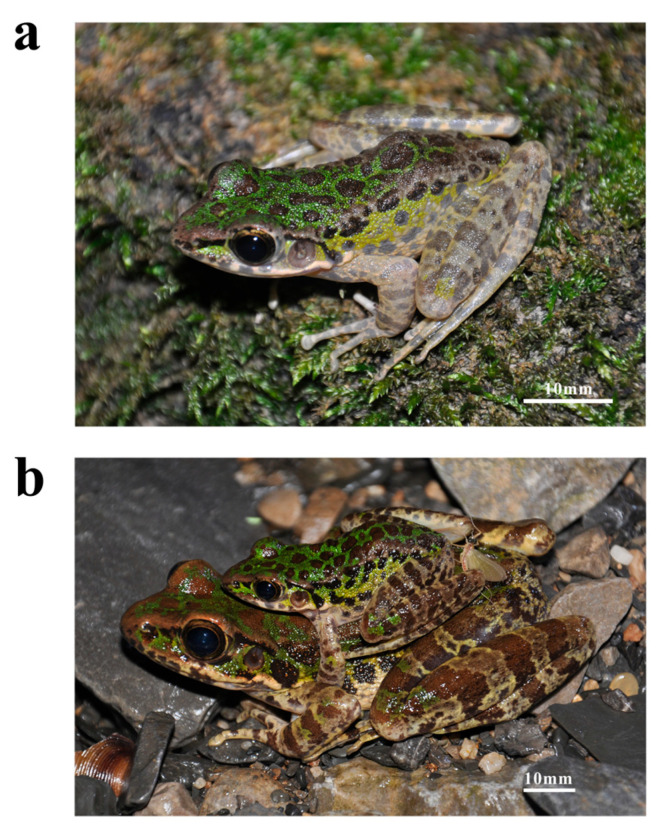
Photographs of male (**a**) and female ((**b**); below) of *O*. *schmackeri*.

The piebald odorous frog *O*. *schmackeri* (Figure 1), widely distributed across Asian biodiversity hotspots from China to Southeast Asia, is an ideal model for studying host-microbiota interactions, its broad environmental tolerance is linked to exceptional gut and skin microbial diversity [65,66,67]. Decoding its microbiota-host dynamics can offer insights into physiological adaptations to diverse habitats and environmental influences on microbial functions, as seen in pesticide degradation pathways of sympatric amphibians [15,19,23]. Integrating sex-specific analyses with biogeographic variables may uncover new microbial plasticity axes in this pivotal species. To explore these dynamics in the ecologically sensitive *O. schmackeri*, we used 16S rRNA gene amplicon sequencing to measure microbial community responses to three key drivers: host sex, tissue specificity (gut vs. skin), and environmental gradients. This dual-axis investigation addresses two pivotal questions: how intrinsic (sex and animal tissues) and extrinsic (habitat) factors interactively shape microbial assemblages, and what metabolic pathways (predicted via PICRUSt2) drive microbiota-mediated environmental adaptation. By combining taxonomic and functional analyses, this study creates a framework for identifying microbial biomarkers for habitat monitoring and devising species-specific protection strategies.

## 2. Materials and Methods

The overall experimental workflow of this study is illustrated in Figure S1, primarily involving sample collection, data processing, and result presentation.

### 2.1. Sample Collection

A total of 19 adult *O. schmackeri* frogs (10 females and 9 males; Figure 1) were captured between July and August 2024 from a 10 m stream transect in Gaojiayan Town, Hubei Province, China (30°36′06″ N, 111°02′56″ E; elevation 274 m). The sampling site exhibited stable hydrological conditions, with the water temperature consistently maintained at 22.7 °C. All specimens were collected nocturnally between 18:00 and 24:00 to control for circadian rhythm effects. Sexual dimorphism was observed in snout-vent length (SVL), with females (81.05 ± 6.89 mm) being significantly larger than males (42.51 ± 2.36 mm). Each individual was rinsed three times with 50 mL of sterile water to remove transient environmental microbes. Resident skin microbiota were collected using standardized swabbing procedures (three passes each on dorsal, lateral, and ventral surfaces) with sterile nylon-flocked swabs (SHX1-9 and SHC1-10 represented male and female skin microbial samples, respectively). Following euthanasia with buffered 1% MS-222 (tricaine methanesulfonate; Sigma-Aldrich Corporation, St. Louis, MO, USA), the entire intestinal tracts were aseptically dissected and stored separately (GHX1-9 and GHC1-10 represented male and female gut microbial samples). Four replicate water samples (W1-4) were collected by filtering through 0.22 μm membranes (47 mm diameter; Millipore Corporation, Billerica, MA, USA). Quadruplicate soil cores (30 cm × 30 cm × 10 cm depth, spaced 10 m apart) were obtained (TR1-4) from riparian zones adjacent to frog microhabitats. All biological and environmental samples were immediately flash-frozen in liquid nitrogen and stored at −80 °C until DNA extraction. All sampling procedures followed the ARRIVE guidelines for in vivo experimentation and were approved by the Institutional Animal Care and Ethics Committee of Henan Normal University, complying with China’s national ethical and legal standards.

### 2.2. DNA Extraction and 16S Amplicon Library Preparation

Genomic DNA was extracted using the TGuide S96 Magnetic Soil/Stool DNA Kit (Tiangen Biotech (Beijing) Co., Ltd., Beijing, China). Briefly, 0.25–0.5 g of sample was homogenized in a 2 mL tube with 500 μL Buffer SA, 100 μL Buffer SC, and 0.25 g grinding beads, using either vortexing for 15 min or a TGrinder H24 homogenizer (6 m/s, 2 cycles of 30 s with 30 s intervals). After centrifugation at 12,000 rpm for 1 min, 500 μL supernatant was transferred to a new tube, mixed with 200 μL Buffer SH, vortexed, and incubated at 4 °C for 10 min. Following recentrifugation (12,000 rpm, 3 min), the supernatant was transferred again, and 500 μL Buffer GFAP was added, mixed by inverting, followed by 10 μL MagAttract Suspension GSP1 (Qiagen, Hilden, Germany). After 5 min shaking, the tube was placed on a magnetic stand for 30 s and the supernatant discarded. The beads were washed with 700 μL Buffer RDP and twice with 700 μL Buffer PWDP, removing supernatant by magnetic aspiration after each wash. After air-drying for 5–10 min at room temperature, 50–100 μL Buffer TB was added and incubated at 56 °C for 5 min with shaking. Finally, the tube was placed on the magnetic stand for 2 min, and the purified DNA was transferred to a 96-well plate for storage.

DNA quantity was assessed via electrophoresis on a 1.8% agarose gel, while the concentration and purity were measured using a NanoDrop 2000 UV-Vis spectrophotometer (Thermo Scientific, Wilmington, NC, USA). DNA concentrations were standardized to 5–50 ng/μL to ensure consistent PCR amplification efficiency. To investigate microbial community diversity and composition, the V3–V4 hypervariable region of the 16S rRNA gene was amplified using the primers 338F (ACTCCTACGGGAGGCAGCA) and 806R (GGACTACHVGGGTWTCTAAT), each appended with Illumina-specific index sequences at the 5′ end to facilitate multiplexed sequencing. The PCR reaction mixture (total volume 20 μL) contained 5–50 ng of DNA template, 0.3 μL of each primer, 5 μL of KOD FX Neo Buffer, 2 μL of dNTPs, 0.2 μL of KOD FX Neo DNA polymerase (Toyobo, Osaka, Japan), and ddH_2_O. The thermocycling program included an initial denaturation at 95 °C for 5 min, followed by 20 cycles of denaturation (95 °C, 30 s), annealing (50 °C, 30 s), and extension (72 °C, 40 s), with a final extension at 72 °C for 7 min. Amplified PCR products were purified using the Omega DNA Purification Kit (Omega Bio-Tek, Norcross, GA, USA) and quantified using the Qsep-400 system (BiOptic, New Taipei City, Taiwan). The purified amplicon library was sequenced using paired-end 2 × 250 bp chemistry on an Illumina Novaseq 6000 platform (Beijing Biomarker Technologies Co., Ltd., Beijing, China) to generate high-throughput data for downstream microbial community analysis.

### 2.3. Data Standardization and Analysis

Raw DNA sequences were processed using PEAR 0.9.6 [68] for primer trimming and paired-end read merging. Subsequent demultiplexing was performed based on unique barcode identifiers. A stringent quality control pipeline was applied, involving the removal of low-quality reads using the DADA2 package [69] with default settings, followed by chimera elimination via consensus filtering in QIIME2 [70]. Qualified sequences were clustered into amplicon sequence variants (ASVs) using USEARCH (version 10.0) at a similarity threshold of 100%. Taxonomy annotation of ASVs was performed using the Naive Bayes classifier in QIIME2 [70] against the SILVA database (release 138.1) [71] with a confidence threshold of 70%. QIIME2 was used to filter ASVs with a relative abundance < 1% in all samples and retained taxonomic units with relative abundance >1% in all groups. A Wilcoxon rank-sum test with Benjamini–Hochberg False Discovery Rate (BH-FDR) correction was conducted to compare environmental microbial community composition (soil/water) at the phylum and genus levels. The Kruskal–Wallis test with BH-FDR correction was used to compare host microbial community composition. If significant differences (*p* < 0.05) existed among multiple groups, the Nemenyi post hoc test method was conducted for pairwise comparisons, including sex-specific gut and skin microbiota (GHC vs. GHX; SHC vs. SHX) and tissue-specific gut and skin microbiota (GHX vs. SHX; GHC vs. SHC). Gene function was predicted and assigned them to corresponding pathways using Phylogenetic Investigation of Communities by Reconstruction of Unobserved States 2 (PICRUSt2), based on the Kyoto Encyclopedia of Genes and Genomes (KEGG) database [72,73]. Results were presented as mean ± standard deviation. Significant differences in functional abundance between groups (tissue-specific: GHC vs. SHC, GHX vs. SHX; sex-specific: GHC vs. GHX; SHC vs. SHX) were analyzed using the Fisher exact test in STAMP [74].

Species diversity within individual samples was assessed using QIIME2, with alpha diversity indices (Chao1 index and Shannon index) calculated to quantify microbial richness and diversity. Differences in alpha diversity indices among sample groups were evaluated using a Wilcoxon rank-sum test at the ASV level. Non-Metric Multi-Dimensional Scaling (NMDS) was employed to visualize bacterial community composition based on Weighted UniFrac distances [75], which account for both phylogenetic relatedness and abundance differences between samples. The stress value was used to evaluate the ordination reliability. Samples positioned closer together on NMDS plots exhibited higher compositional similarity. Permutational multivariate analysis of variance (PERMANOVA) and analysis of similarities (ANOSIM) were conducted at the ASV level to test for significant structural differences in β-diversity among groups.

Linear Discriminant Analysis (LDA) Effect Size (LEfSe) was used to identify biomarkers that exhibited statistically significant differences among species groups. A non-parametric Kruskal–Wallis (KW) test was used to detect features with significant differences across sample groups. LEfSe analysis further estimated the effect sizes of these intergroup differences using LDA scores, with a log LDA score > 4.5 set as the threshold for identifying discriminative features [76]. This approach allowed for the prioritization of features that contributed most significantly to the observed differences between groups.

### 2.4. Correlation Analysis of O. Schmackeri Gut and Skin Microbiota

We selected *O. schmackeri* gut (GH: GHX 1–9 and GHC 1–10) and skin (SH:SHX 1–9 and SHC 1–10) microbiota with relative abundances >1% to investigate their correlations (GH vs. SH). To investigate the associations between microbiota from different animal tissues within the same host, we performed a cross-taxonomic group correlation analysis between the GH and the SH groups. We used the package R ‘Vegan’ (version 4.5.2) to calculate correlations between gut and skin microbiota [77]. We used the spearman correlation coefficient (|r| > 0.1, *p* < 0.05) to determine whether significant correlations existed between phyla and between genera [78]. Finally, we used package R ‘ggplot2’ to construct correlation heatmaps for visualizing relationships between phyla and phyla or genus and genus [79]. LEfSe analysis (LDA > 4.5) was used to identify biomarker taxa that significantly differentiated sample groups (GH vs. SH).

In this study, all statistical significances were defined at * *p* < 0.05, ** *p* < 0.01, and *** *p* < 0.001 for pairwise comparisons.

## 3. Results

### 3.1. Microbial Community Composition and Differences in the Habitat of O. schmackeri

**Figure 2:**
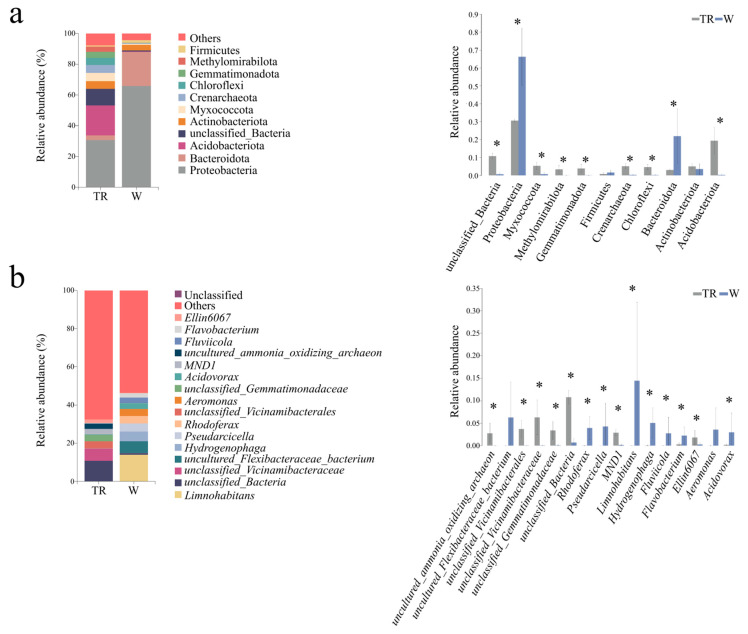
The relative abundances of environmental microbiota (soil and water) at the phylum level (left panel of (**a**)) and genus level (left panel of (**b**)). Only taxa with relative abundances > 1% (at both phylum and genus levels) are displayed for each sample in the environmental microbiota. Each color represents a taxonomic group at the phylum or genus level, with names labeled on the right side of the figure (left panels of (**a**,**b**)). The color for ‘Other’ represents all phyla and genera (with relative abundance < 1%), whose names are not shown in the plots; compositional comparisons of environmental microbiota at the phylum level (right panel of (**a**)) and genus level (right panel of (**b**)) were analyzed using the Wilcoxon rank-sum test with BH-FDR correction. Statistical significances were defined at * *p* < 0.05 for pairwise comparisons.

Environmental microbiota exhibited distinct compositional profiles at the phylum level (relative abundance > 1%; Figure 2a). Soil communities were predominantly composed (relative abundance > 10%) of Proteobacteria (30.63%) and Acidobacteriota (19.50%). In contrast, water microbiota was primarily dominated by Proteobacteria (65.88%) and Bacteroidota (22.11%). Among the predominant phyla (relative abundance > 10%), most exhibited significant differences between soil and water samples (*p* < 0.05; Figure 2a), while only Firmicutes and Actinobacteriota belonging to the non-dominant phyla did not differ significantly (*p* > 0.05; Figure 2a). At the genus level (with relative abundance > 1%), dominant genera were defined as those with a relative abundance exceeding 5% (Figure 2b), In soil, the dominant genus was *unclassified_Vicinamibacteraceae* (6.34%), whereas water communities were mainly represented by *Limnohabitans* (13.94%), *uncultured_Flexibacteraceae_bacterium* (6.33%), and *Hydrogenophaga* (5.06%). Of all the microbial genera, only *uncultured_Flexibacteraceae_bacterium* and *Aeromonas* exhibited no significant differences in their relative abundances between soil and water samples (*p* > 0.05; Figure 2b).

Functional profiling identified several dominant functional categories (relative abundance > 5%; Figure S2a), including Metabolism (soil: 78.68 ± 0.24%, water: 77.00 ± 1.03%; *p* > 0.05), Genetic Information Processing (soil: 7.63 ± 0.03%, water: 6.00 ± 0.31%; *p* < 0.01), and Environmental Information Processing (soil: 5.92 ± 0.12%, water: 7.37 ± 0.66%; *p* < 0.05). In addition, several non-dominant functional categories (with relative abundance < 5%), including Human Diseases (soil: 2.92 ± 0.08%, water: 3.76 ± 0.18%, *p* < 0.01) and Organismal Systems (soil: 1.38 ± 0.01%, water: 1.62 ± 0.06%, *p* < 0.01) in the KEGG Level 1 categories, showed significant differences between soil and water microbial communities. At the secondary functional categories, 18 functional pathways (with relative abundance > 1%; Figure S2b) were detected. Among these, Global and overview maps (soil: 42.43 ± 0.19%, water: 40.38 ± 0.76%; *p* < 0.05), Carbohydrate metabolism (soil: 8.42 ± 0.10%, water: 8.06 ± 0.10%; *p* < 0.01), and Amino acid metabolism (soil: 7.38 ± 0.04%, water: 7.87 ± 0.11%; *p* < 0.01) were the dominant pathways (with relative abundance > 5%). In addition, non-dominant pathways (with relative abundance < 5%) also showed significant differences between soil and water microbiota (e.g., Energy metabolism: soil: 4.58 ± 0.02%, water: 4.23 ± 0.02%; *p* < 0.001 and Membrane transport: soil: 3.15 ± 0.04%, water: 4.09 ± 0.38%; *p* < 0.05).

### 3.2. Composition and Functional Profiles of Host-Associated Microbiota in O. schmackeri

**Figure 3:**
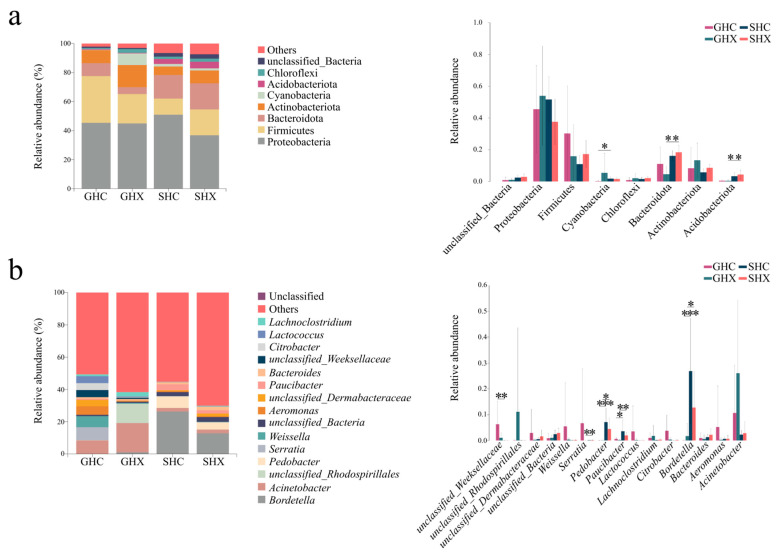
The relative abundances of gut (GHC and GHX) and skin (SHC and SHX) microbiota from female and male *Odorrana schmackeri* are shown at the phylum level (left panel of (**a**)) and genus level (left panel of (**b**)). Only taxa with relative abundances > 1% (at both phylum and genus levels) are displayed for each sample in the frog microbiota. Each color represents a taxonomic group at the phylum or genus level, with names labeled on the right side of the figure (left panels of (**a**,**b**)). The color for ‘Other’ represents all phyla and genera (with relative abundance < 1%), whose names are not shown in plots; compositional comparisons of gut and skin microbiota at the phylum level (right panel of (**a**)) and genus level (right panel of (**b**)) were analyzed using the Wilcoxon rank-sum test with BH-FDR correction. Statistical significances were defined at * *p* < 0.05, ** *p* < 0.01, and *** *p* < 0.001 for pairwise comparisons.

The gut and skin microbiota of *O. schmackeri* were primarily composed of Proteobacteria, Firmicutes, Bacteroidota and Actinobacteriota at the phylum level (relative abundance > 10%; Figure 3a). Proteobacteria was the most abundant phylum in both gut (females: 45.36%; males: 44.93%) and skin microbiota (females: 50.98%; males: 36.78%). In the gut, Firmicutes ranked second (females: 32.20%; males: 20.26%), followed by Actinobacteriota in females (8.95%) and males (15.21%). In contrast, Bacteroidota constituted the second most abundant phylum in skin microbiota (females: 16.22%; males: 17.98%), with Firmicutes being the third (females: 11.11%; males: 17.81%). Statistical analysis revealed distinct tissue-specific patterns in phylum-level abundance (Figure 3a), including Cyanobacteria (female gut: 0.23%, female skin: 1.72%; *p* < 0.05), Bacteroidota (male gut: 4.75%, male skin: 17.98; *p* < 0.01), and Acidobacteriota (male gut: 0.34%, male skin: 4.54; *p* < 0.01).Notably, the relative abundances of both dominant and non-dominant phyla showed no significant sexual differences (*p* > 0.05; Figure 3a). At the genus level (relative abundance > 1%, Figure 3b), dominant genera were defined as those exceeding 5% in relative abundance. *Acinetobacter* was the most abundant genus in the gut microbiota of both female (8.35%) and male (18.19%). In females, the dominant gut community also included *Serratia* (8.04%), *Weissella* (6.84%), and *Aeromonas* (5.25%). In males, the gut microbiota exhibited a high relative abundance in *unclassified_Rhodospirillales* (11.91%). In skin microbiota, *Bordetella* was the predominant genus in both females (26.34%) and males (12.83%). Female skin also characterized by *Pedobacter* (7.13%) as an additional dominant genus, whereas no other genus exceeded 5% relative abundance in male skin. Statistical analysis confirmed significant intergroup differences at the genus level (Figure 3b). Some dominant genera (e.g., *Bordetella* and *Pedobacter*) showed significant differences between animal tissues (*p* < 0.05), as did some non-dominant genera (e.g., *Paucibacter* and *unclassified_Weeksellaceae*; *p* < 0.05). In contrast, no sex-specific differences were observed in either dominant (with relative abundance > 5%) or non-dominant (with relative abundance < 5%) genera.

Metabolic pathways represented the most abundant functional category in both gut and skin microbiota of *O. schmackeri*, accounting for 77.65 ± 1.12% (GHC), 77.79 ± 3.30% (GHX), 77.82 ± 0.55% (SHC), and 78.52 ± 0.27% (SHX) of annotated gene functions (Figure S3a). Other dominant functional categories (relative abundance > 5%) included Genetic Information Processing (GHC: 7.07 ± 0.93%, GHX: 7.66 ± 2.46%, SHC: 6.89 ± 0.26%, and SHX: 7.13 ± 0.32%) and Environmental Information Processing (GHC: 7.54 ± 0.98%, GHX: 6.85 ± 0.53%, SHC: 7.04 ± 0.45%, and SHX: 6.59 ± 0.27%). Significant sex-related differences in the metabolic (SHC: 77.82 ± 0.55%, SHX: 78.52 ± 0.27%; *p* < 0.01), human diseases (SHC: 3.29 ± 0.19%, SHX: 3.00 ± 0.21%; *p* < 0.01), Cellular Processes (SHC: 3.40 ± 0.22%, SHX: 3.23 ± 0.08%; *p* < 0.05), and Environmental Information Processing (SHC: 7.04 ± 0.45%, SHX: 6.59 ± 0.27%; *p* < 0.05) functions were detected in skin microbiota (SHC vs. SHX), while a significant tissue-related difference in human diseases (GHC: 2.95 ± 0.23%, SHC: 3.29 ± 0.19%; *p* < 0.01) function was also detected in gut microbiota (GHC vs. SHC). No other significant tissue- and sex- related differences were observed among the functional categories (*p* > 0.05). At KEGG level-2 (Figure S3b), the most abundant pathways were Global and overview maps (GHC: 41.25 ± 0.66%, GHX: 41.17 ± 2.02%, SHC: 41.27 ± 0.46%, and SHX: 41.75 ± 0.32%), Carbohydrate metabolism (GHC: 9.32 ± 0.68%, GHX: 8.62 ± 0.85%, SHC: 8.41 ± 0.27%, and SHX: 8.80 ± 0.30%), and Amino acid metabolism (GHC: 6.95 ± 0.57%, GHX: 7.07 ± 1.11%, SHC: 7.40 ± 0.21%, and SHX: 7.39 ± 0.27%). Significant tissue- and sex-related differences were observed in KEGG Level-2 pathway (*p* < 0.05), including: Amino acid metabolism (GHC: 6.95 ± 0.57% vs. SHC: 7.40 ± 0.21%; *p* < 0.05), Carbohydrate metabolism (GHC: 9.32 ± 0.68% vs. SHC: 8.41 ± 0.27%; *p* < 0.01; SHC: 8.41 ± 0.27% vs. SHX: 8.80 ± 0.30%; *p* < 0.05), Cellular community—prokaryotes (GHC: 1.55 ± 0.24% vs. SHC: 1.92 ± 0.30%; *p* < 0.05), Energy metabolism (GHC: 4.09 ± 0.24% vs. SHC: 4.51 ± 0.18%; *p* < 0.001; SHC:4.51 ± 0.18% vs. SHX: 4.36 ± 0.07%; *p* < 0.05), Metabolism of terpenoids and polyketides (GHC: 1.13 ± 0.09% vs. SHC: 1.22 ± 0.02%; *p* < 0.05), Global and overview maps (SHC: 41.27 ± 0.46% vs. SHX: 41.75 ± 0.32%; *p* < 0.05), and Replication and repair (SHC: 2.51 ± 0.06% vs. SHX: 2.60 ± 0.07%; *p* < 0.05).

### 3.3. Comparison of Diversity Differences and Biomarkers in the Environmental and Host-Associated Microbiota

**Figure 4:**
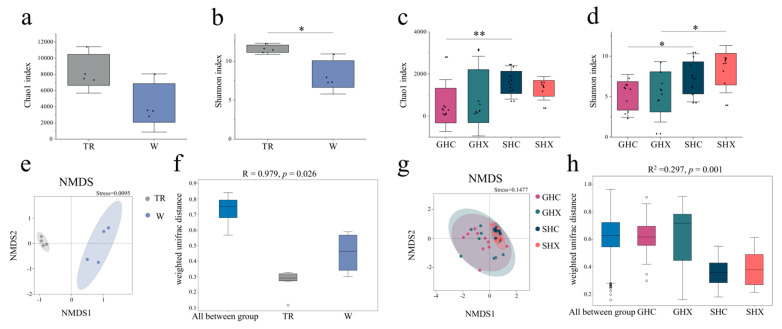
This figure displays comparisons of Chao1 (**a**,**c**) and Shannon (**b**,**d**) indices in soil (TR) and water (W) microbiota from the environment (**a**,**b**), and in frog gut (GHC and GHX) and skin (SHC and SHX) microbiota (**c**,**d**), which analyzed using the Wilcoxon rank-sum test; β-Diversity of bacterial community structure is shown via NMDS plots: the panel (**e**) shows environmental bacterial communities (soil and water microbiota), while panel (**f**) illustrates the result of the ANOSIM test; the panel (**g**) illustrates frog bacterial communities (gut and skin microbiota) and the panel (**h**) shows the result of PERMANOVA test. Statistical significances were defined at * *p* < 0.05, ** *p* < 0.01 for pairwise comparisons.

Microbial α-diversity analysis revealed significant differences in bacterial richness (Chao1 index) and diversity (Shannon index) across both environmental microbiota and host-associated communities (Figure 4a–d). While bacterial richness did not differ significantly between soil and water microbiota (*p* > 0.05), soil communities exhibited significantly higher diversity (Figure 4a,b, *p* < 0.05). In host-associated microbiota, no significant sex-based differences in richness or diversity (both *p* > 0.05) were detected in either gut (GHC vs. GHX) or skin (SHC vs. SHX) samples (Figure 4c,d). However, tissue-specific variations were evident, with both richness and diversity being significant higher in female gut than skin microbiota (GHC vs. SHC; both *p* < 0.05). In males, although richness did not differ between gut and skin microbiota (GHX vs. SHX; *p* > 0.05), diversity was significantly higher in gut microbiota (GHX vs. SHX; *p* < 0.05).

NMDS ordinations based on Weighted UniFrac distances showed clear separations of bacterial communities in environmental samples (ANOSIM, *R* = 0.979, *p* = 0.026, Figure 4e,f), as well as between gut and skin microbiota of *O. schmackeri* (PERMANOVA, *R*^2^ = 0.297, *p* = 0.001, Figure 4g,h). Sex did not significantly influence community composition in gut (GHC vs. GHX: ANOSIM, *R* = 0.021, *p* = 0.271) or skin (SHC vs. SHX: ANOSIM, *R* = 0.124, *p* = 0.083). In contrast, strong compartmentalizations were observed between gut and skin microbiota in both males (GHX vs. SHX: ANOSIM, *R* = 0.454, *p* = 0.002) and females (GHC vs. SHC: ANOSIM, *R* = 0.590, *p* = 0.001).

**Figure 5:**
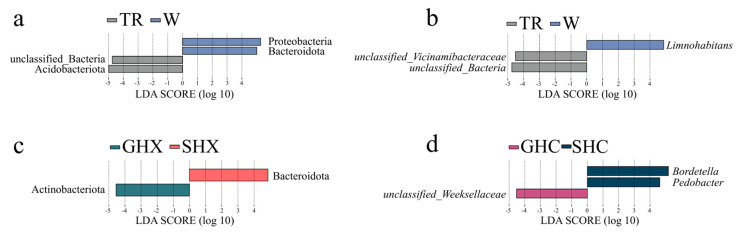
LDA scores (>4.5) identified biomarkers at the phylum and genus levels in soil and water microbiota from environment (**a**,**b**), as well as in gut and skin microbiota of female and male frogs (**c**,**d**).

LEfSe analysis identified distinct microbial biomarkers in both environmental and *O. schmackeri*-associated communities at the phylum and genus levels (LDA > 4.5; Figure 5). In environmental samples, Proteobacteria (LDA = 5.26, *p* = 0.021) and Bacteroidota (LDA = 5.01, *p* = 0.021) were identified as characteristic biomarkers for water communities, while Acidobacteriota (LDA = 4.98, *p* = 0.021) was specific to soil (Figure 5a). At the genus level, *Limnohabitans* (LDA = 4.87, *p* = 0.014) was a biomarker for water systems, and *unclassified_Vicinamibacteraceae* (LDA = 4.51, *p* = 0.021) was associated with soil (Figure 5b). Among host-associated microbiota, Bacteroidota (LDA = 4.86, *p* < 0.001) was a phylum-level biomarker for male skin, whereas Actinobacteriota (LDA = 4.55, *p* = 0.047) specifically characterized the male gut (Figure 5c). No significant phylum-level biomarkers were detected in female samples (*p* > 0.05). In contrast, at the genus level, female skin microbiota was distinguished by *Bordetella* (LDA = 5.18, *p* < 0.001) and *Pedobacter* (LDA = 4.63, *p* < 0.001), and female gut microbiota by *unclassified_Weeksellaceae* (LDA = 4.53, *p* < 0.001). No genus-level biomarkers were identified in male samples (Figure 5d).

### 3.4. Microbial Correlations and Tissue-Specific Biomarkers in O. schmackeri

**Figure 6:**
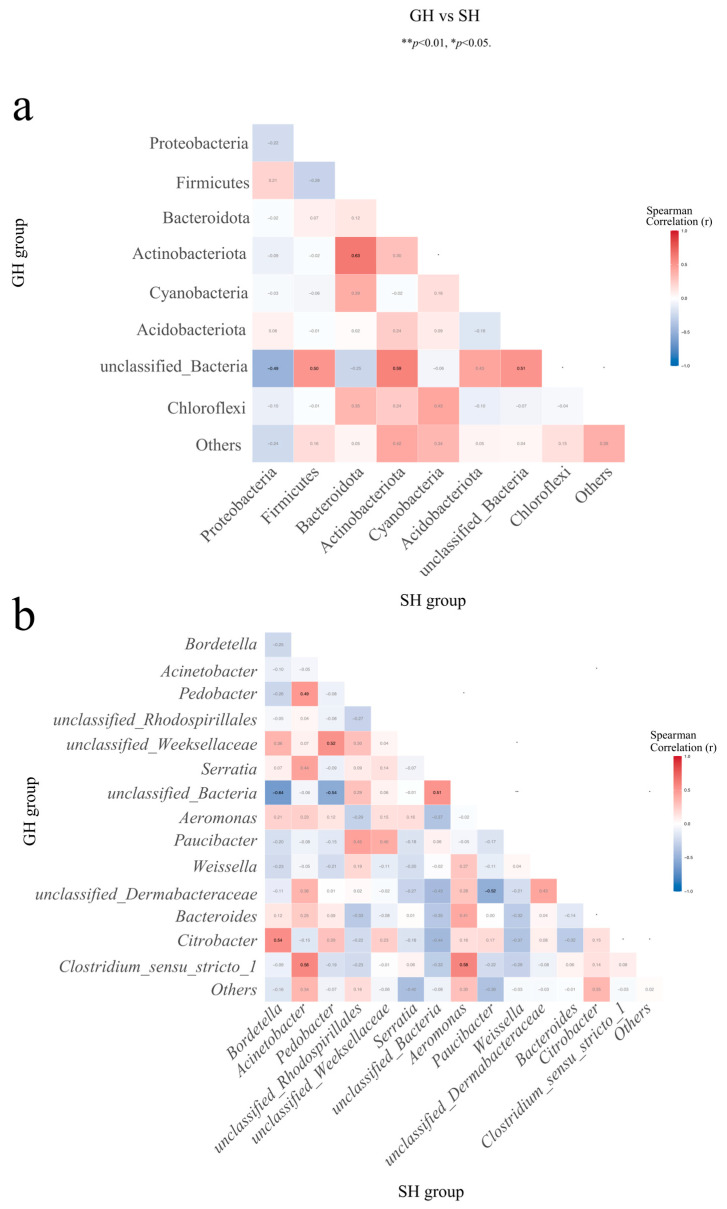
Heatmaps showing the correlations between gut (GH) and skin (SH) microbiota. The labels on the horizontal and vertical axes indicate names of species and source groups. the color gradients represent the changes in Spearman correlation coefficients between samples, and correlation data are listed on the right. Data with darker font colors indicate significant correlations between samples or indicated by significance thresholds. Statistical significances were defined at * *p* < 0.05, ** *p* < 0.01 for pairwise comparisons. (**a**) Correlations between gut (GH) and skin (SH) microbial communities at the phylum level; (**b**) Correlations between gut (GH) and skin (SH) microbial communities at the genus level.

Spearman correlation analysis revealed several significant relationships between microbial taxa across gut and skin microbiota (Tables S1 and S2). At the phylum level, a strong positive correlation was observed between gut Actinobacteriota and skin Bacteroidota (r = 0.63, *p* < 0.01; Figure 6a), whereas a moderate negative correlation was found between unclassified_Bacteria in the gut and skin Proteobacteria (r = −0.49, *p* < 0.05). No other significant correlations were observed among the dominant bacteria phyla. Similar analyses at the genus level revealed additional associations. A moderate positive correlation was identified between gut *Pedobacter* and skin *Aeromonas* (r = 0.47, *p* < 0.05; Figure 6b), as well as between gut *Pedobacter* and skin *Acinetobacter* (r = 0.49, *p* < 0.05). Beyond these dominant taxa, several significant correlations were also found between dominant and non-dominant groups, as well as non-dominant bacterial taxa at both taxonomic levels (Tables S1 and S2; |r| > 0.4, *p* < 0.05).

**Figure 7:**
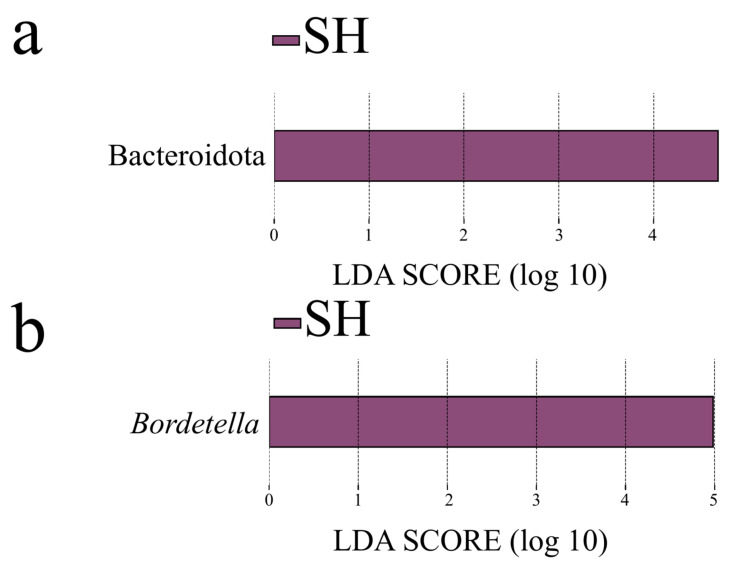
LDA scores (>4.5) identified biomarkers at the phylum (**a**) and genus (**b**) levels between *Odorrana schmackeri* gut (GH) and skin (SH) microbiota.

LEfSe analyses further identified tissue-specific microbial biomarkers in *O. schmackeri* (LDA > 4.5; Figure 7a). Bacteroidota was identified as a significant phylum-level biomarker for the skin microbiota (LDA = 4.68, *p* < 0.001; Figure 7a), while no phylum-level biomarkers were detected for the gut. At the genus level, *Bordetella* was a specific biomarker for the skin microbiota (LDA = 4.98, *p* < 0.001; Figure 7b) while no significant genus-level biomarkers in the gut microbiota.

## 4. Discussion

In this study, we identified significant differences in environmental (soil vs. water) microbiota composition and a strong effect of tissue type on host-associated microbiota, with limited sexual dimorphism. In line with this, diversity analyses (Shannon, Chao1, beta-diversity; Figure 4) and LEfSe results (Figure 5c,d) confirmed tissue specificity as the primary driver of host-associated community structure. Functional profiling, however, revealed a predominance of metabolic pathways and uncovered a latent sexual dimorphism not seen in composition. Significant functional differences were observed between environments (Figure S2) and, within hosts, for both tissue type and sex at primary and secondary KEGG pathway levels (Figure S3), indicating sex-specific adaptations at the functional level.

We found that Proteobacteria was the dominant phylum in both environmental microbiota (soil/water) and host-associated microbial communities (gut/skin) of *O. schmackeri*, a pattern consistent with observations in other amphibians. For instance, this Proteobacteria predominates in the skin microbiota of *Quasipaa spinosa* [18], *Pseudacris crucifer* [21], *Hoplobatrachus rugulosus* [56], and wild *Rana dybowskii* [80], as well as the gut microbiota of *Lithobates pipiens* tadpoles [20], *Bufo gargarizans* [81], and unhealthy *R. dybowskii* [82]. However, microbial dominance in amphibians is not universal and exhibits species- and context-dependent variability. For example, gender-induced differences in the most abundant phylum—the gut microbiota of *O*. *tormota* shifts from Bacteroidetes-dominant in females to Firmicutes-dominant in males [62], while survival habitat caused differences in the most dominant phylum—natural versus farmland populations of *Fejervarya limnocharis* show a reversal in gut phylum composition (Bacteroidetes: 35.43% vs. Firmicutes: 46.02%) [36]. Unique dominance patterns are also observed, such as Verrucomicrobia in the skin microbiota of *Plethodon cinereus* [83]. Despite this variability, amphibian microbiomes exhibit a balance between compositional plasticity and functional stability. While Environmental pressures drive shifts in microbial community structure, such as habitat-induced changes in skin microbiota diversity and disease resistance [29,84,85,86,87], gut microbiota often retain conserved metabolic functions even when community composition changes [29,88]. Seasonal dynamics further illustrate this interplay, skin microbiota in *Rana sylvatica* show elevated Acidobacteria and Actinobacteria during summer and autumn compared to spring [85]. These findings highlight a dual paradigm in amphibian microbiomes, phylum-level flexibility allows adaptation to environmental pressures, while core functional stability ensures physiological homeostasis.

The functional dichotomy of amphibian-associated microbial phyla highlights the complexity of host–symbiont interactions. Proteobacteria exhibits dual ecological roles, contributing to host metabolism, oligotrophic adaptation, and vitamin biosynthesis [52,89,90], while harboring opportunistic pathogens (*Yersinia*, *Pseudomonas*, *Aeromonas*) linked to gastrointestinal and systemic infections [35,51,82]. *Firmicutes* and *Bacteroidota* further exemplify functional synergy in enhancing host fitness through complementary mechanisms. Elevated Firmicutes/Bacteroidota ratios optimize caloric harvest from dietary substrates [36,89], while Firmicutes-dominated communities acidify cutaneous microenvironments, thereby inhibiting pathogen colonization [18,91]. Meanwhile, Bacteroidota facilitates immune system maturation and polysaccharide digestion [92,93]. This paradox underscores the ecological complexity of amphibian microbiomes, where microbial taxonomy alone inadequately predicts functional outcomes. Instead, functional roles are context-dependent, shaped by host physiology, environmental pressures, and microbial interactions.

Microbial community differentiation at finer taxonomic resolutions reflects intricate interactions between host biology and ecological variables. Genus-level compositional variations across sexes, host tissues, and environmental niches are shaped from multifactorial influences, including seasonal habitat use and foraging strategies [15,47], dietary niche partitioning [94,95], disease-mediated microbiome remodeling [18,96], and species-specific microbial signatures [97]. Key genera exhibit niche-adaptive functionalities that align with their ecological contexts. For instance, *Acinetobacter* demonstrates dual roles in cutaneous chytridiomycosis resistance against *Batrachochytrium dendrobatidis* [98] and intestinal vitamin metabolism [99]. Notably, *Acinetobacter* serves as a paradigm of microbial functional ambivalence. While certain strains pose zoonotic risks, such as causing cutaneous necrosis or pneumonia [96,100], others exhibit protective roles through anti-fungal activity against *Batachochytrium dendrobatid* and biofilm-mediated microbiome stabilization [51,101]. *Limnohabitans* have the function of utilizing algal exudates from the water environment [102]. This taxonomic resolution-specific variation underscores the functional plasticity of microbial systems in response to hierarchical ecological pressures. Such plasticity spans scales of organization, from cellular metabolic processes to ecosystem-level nutrient cycling, highlighting the dynamic interplay between microbial communities and their environments.

Metabolic pathways dominated the functional signature of all analyzed niches, accounting for over 75% of annotated gene functions in environmental (soil and water) microbial communities (Figure S2a), as well as in both gut and skin microbiota of *O. schmackeri* (Figure S3a). This metabolic prevalence is consistent with previous findings of universal energy acquisition strategies in amphibian microbiomes [16,56]. The metabolic enrichment observed in environmental microbiota suggests functional transmission across niches, likely mediated by host–environment microbial exchange [29,59]. The KEGG Level 1 (Figure S3a) and Level 2 (Figure S3b) pathways demonstrated tissue specificity and sexual dimorphism, potentially attributable to differential microenvironmental exposure. Notably, our results were similar to studies associating gut functional sex differences with trophic niche partitioning [60]. While Shannon index analysis demonstrated significant bacterial diversity between gut and skin microbiota of male frog microbiota (Figure 4d; *p* < 0.05), KEGG enrichment comparisons revealed no tissue-driven functional differentiation in primary or secondary metabolic/physiological pathways. This dichotomy suggests tissue-specific community structuring without corresponding functional specialization in male *O. schmackeri*.

Our analysis revealed no significant intersexual differences in α-diversity metrics (richness/diversity) of *O. schmackeri* gut or skin microbiota (Figure 4c,d), a finding consistent with observations in *R. sylvatica* skin communities [85] but contrasting with the sexually dimorphic patterns reported in *Pelophylax perezi* [61]. Variations in α-diversity are predominantly influenced by genotype-driven microbial community assembly [16], habitat-specific microbial recruitment [97], and developmental restructuring of the microbiome [103]. While sex-specific α-diversity patterns remain inconsistent across amphibian species [60,62,104], potential sampling limitations in prior studies (e.g., *R. sylvatica* female sample size) necessitate cautious interpretation. Our results underscore the importance of standardized, phylogenetically broad investigations to resolve whether sexual dimorphism in amphibian microbiomes reflects species-specific adaptations or broader ecological patterns, and whether observed differences arise from methodological artifacts or genuine biological signals. This ambiguity highlights the complex interplay between host physiology and environmental filters in shaping microbial ecology, demanding integrative meta-analyses across life histories and habitats. Tissue-specific microbial niche partitioning has been recognized as a key factor shaping amphibian microbiome architecture [105,106]. Comparative analysis revealed significant α-diversity divergences between gut and skin microbiota (Figure 4c,d), though male specimens showed comparable richness across niches (Chao1; *p* > 0.05). This tissue-driven microbial stratification reflects broader ecological patterns. Skin microbiota exhibit heightened plasticity and environmental susceptibility due to their direct interface with external habitats [47], particularly with aquatic microbial reservoirs [22,59,107]. Gut microbiota demonstrate greater stability, with dietary influences predominating over geographic effects, as evidenced by consistent profiles within shared habitats [29,36,62]. Hierarchical partitioning of environmental versus trophic influences further clarifies these patterns: for skin microbiota, water-associated microbiota explained 38% of community structure variance [31,54]; for gut microbiota, dietary specialization drove 42% of functional gene variation [95,108]. These findings highlight a tissue–environment–trophic axis that shapes amphibian microbiomes. Skin communities prioritize responsiveness to environmental fluctuations, while gut systems emphasize adaptation to dietary inputs. This dichotomy reflects an evolutionary trade-off between external environmental sensing and internal physiological homeostasis, underscoring the adaptive complexity of host-microbiota interactions in amphibians. The Chao1 and Shannon indices demonstrated significant differences (Figure 4c,d; *p* < 0.05) between the gut and skin microbiota of female *O. schmackeri*, accompanied by multi-scale functional differentiation, highlighting the tissue microenvironment-driven metabolic specialization in female *O. schmackeri*. Instead, environmental filtering appears to be the dominant force shaping these patterns, rather than dietary influences. The functional homogenization observed across host sexes and environmental reservoirs underscores habitat-driven metabolic convergence, a critical adaptation for amphibians inhabiting environments with fluctuating resources.

Our analyses revealed distinct microbial community structures in *O. schmackeri*, shaped predominantly by host tissue type and environmental exposure. Significant compositional segregations between gut and skin microbiota were observed in both sexes (GHC vs. SHC, SHC vs. SHX; ANOSIM: r > 0.45, *p* < 0.01; Figure 4g,h), while no intersexual differentiation emerged within these niches. Hierarchical clustering identified shared habitat and anatomical niche—not host sex—as the primary drivers of microbial assembly. This aligns with broader ecological patterns where environmental filtering and tissue-specific adaptations play a more prominent role in structuring amphibian microbiomes than sexual dimorphism [85,109,110,111]. These findings collectively emphasize that microbial community organization in amphibians is governed by a hierarchy of ecological factors, with tissue-specific functional demands and environmental interactions taking precedence over host sex. Tissue-specificity was confirmed in the LEfSe analysis of skin and gut microbiota across the four groups at high and low altitude [112]. Furthermore, our LEfSe analysis also demonstrated the influence of host tissue-specificity on the microbiota (Figure 5c,d and Figure 7).

Spearman’s correlation analysis revealed several key correlations between the gut and skin microbiota at the phylum (Figure 6a) and genus taxonomic levels (Figure 6b). At the dominant phylum level, we observed a significant positive correlation between Actinobacteriota in the gut microbiota and Bacteroidota in the skin microbiota (r = 0.63, *p* < 0.01; Table S1). We also observed a significant positive correlation at the phylum level between Actinobacteriota in the gut microbiota and Cyanobacteria in the skin microbiota (r = 0.5, *p* < 0.05). Additionally, this correlation pattern was similarly observed at the dominant genus level (Figure 6b; Table S2). Notably, this gut–skin axis demonstrates that the influence of the microbiota on animal tissues is bidirectional [113,114]. Imbalances in the microbiota may affect the microbiota of other animal tissues, resulting in disease/health mechanisms. For instance, the absorption of ultraviolet B light by the skin may improve the gut microbiota, or conversely, gut microbiota imbalances may lead to the development of skin inflammation.

## 5. Conclusions

Our integrated analysis reveals a tripartite interaction among host sex, tissue specificity, and environmental reservoirs in shaping the *O. schmackeri* microbiome architecture, including its compositional diversity, community structure, and function potential. Multilevel analysis demonstrated that soil and water microbiota exhibit differences in composition, diversity, and function, with significant differences also observed among animal tissues. This may be mediated by differential bacterial transmission efficiency across environmental interfaces. The absence of significant differences in gut and skin microbiota composition and diversity between sexes may also be attributed to similar environmental exposure or microbial transmission patterns. However, at finer taxonomic resolutions, most samples exhibited distinct microbial profiles, suggesting that additional factors such as survival behaviors, disease dynamics, and microhabitat use may shape frog microbiota in complex ways. Gene function analysis showed that metabolic processes were the predominant functional signature in *O. schmackeri*, constituting a major portion of annotated gene functions. Notably, environmental microbiota exhibited consistent high enrichment rates for metabolic functions, indicating that frog microbiota may be influenced by functional traits acquired from environmental reservoirs. These findings highlight the intricate interplay between frog microbiota and their environment, emphasizing the importance of ecological context in shaping microbial communities and their functional potential. This study also revealed correlations among microbiota in different ecological niches. These findings can provide insights into synergistic or competitive relationships between microbiota for subsequent research and identify potential links between these interactions and host health.

## Data Availability

The data presented in this study are openly available in NCBI SRA database at http://www.ncbi.nlm.nih.gov/bioproject/1353854 (accessed on 27 November 2025), reference PRJNA1353854.

## Supplementary Materials

The following supporting information can be downloaded at: https://www.mdpi.com/article/10.3390/microorganisms13122725/s1, Figure S1: Flowchart of the overall experimental workflow. Figure S2: gene functional categories predicted from 16S rRNA gene data in environmental soil (TR) and water (W) microbiota are shown at KEGG Level 1 (a) and Level 2 (b) categories, presented as the mean ± SD of relative abundance. Figure S3: gene functional categories predicted from 16S rRNA gene data in the gut (GHC and GHX) and skin (SHC and SHX) microbiota of female and male *Odorrana schmacker* are shown at KEGG Level 1 (a) and Level 2 (b) categories, presented as the mean ± SD of relative abundance. Table S1: Results of correlation coefficients and significance values between gut microbiota and skin microbiota at the phylum taxonomic level. Table S2: Results of correlation coefficients and significance values between gut microbiota and skin microbiota at the genus taxonomic level.

## Abbreviations

The following abbreviations are used in this manuscript:

ANOSIM	Analysis of Similarities
ASV	Amplicon sequence variant
Bd	*Batrachochytrium dendrobatid*
BH-FDR	Benjamini–Hochberg False Discovery Rate
GH	*O. schmackeri* gut
GHC	Female gut of *O. schmackeri*
GHX	Male gut of *O. schmackeri*
KEGG	Kyoto Encyclopedia of Genes and Genomes
KW	Kruskal–Wallis
LDA	Linear discriminant analysis
LEfSe	Linear Discriminant Analysis Effect Size
MS-222	Tricaine methanesulfonate
NMDS	Non-Metric Multi-Dimensional Scaling
OTU	Operational taxonomic unit
PERMANOVA	Permutational multivariate analysis of variance
PICRUSt 2	Phylogenetic Investigation of Communities by Reconstruction of Unob served States 2
SH	*O. schmackeri* skin
SHC	Female skin of *O. schmackeri*
SHX	Male skin of *O. schmackeri*
SVL	Snout-vent length
TR	Soil
W	Water
