# Supplementary figures and images for: Comparative Analysis of Environmental and Host-Associated Microbiome in Odorrana schmackeri (Auran: Ranidae): Insights into Tissue-Specific Colonization and Microbial Adaptation
Source: Microorganisms. 2025 Nov 29;13(12):2725. doi: 10.3390/microorganisms13122725 (PMC12735184; doi:10.3390/microorganisms13122725)

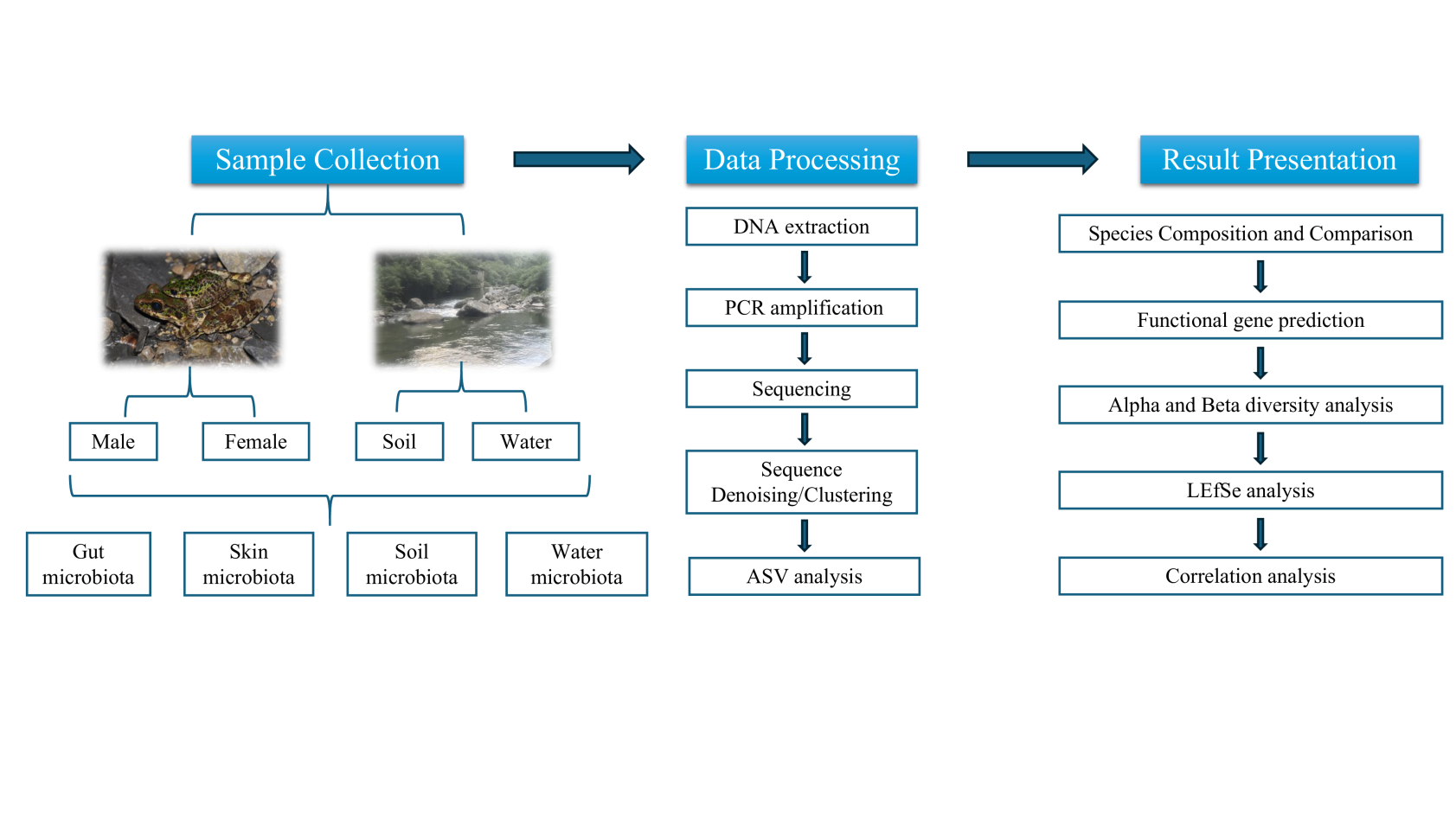

Supplement: Supplementary file 1 [file microorganisms-13-02725-s001.zip › Fig. S1.tiff]

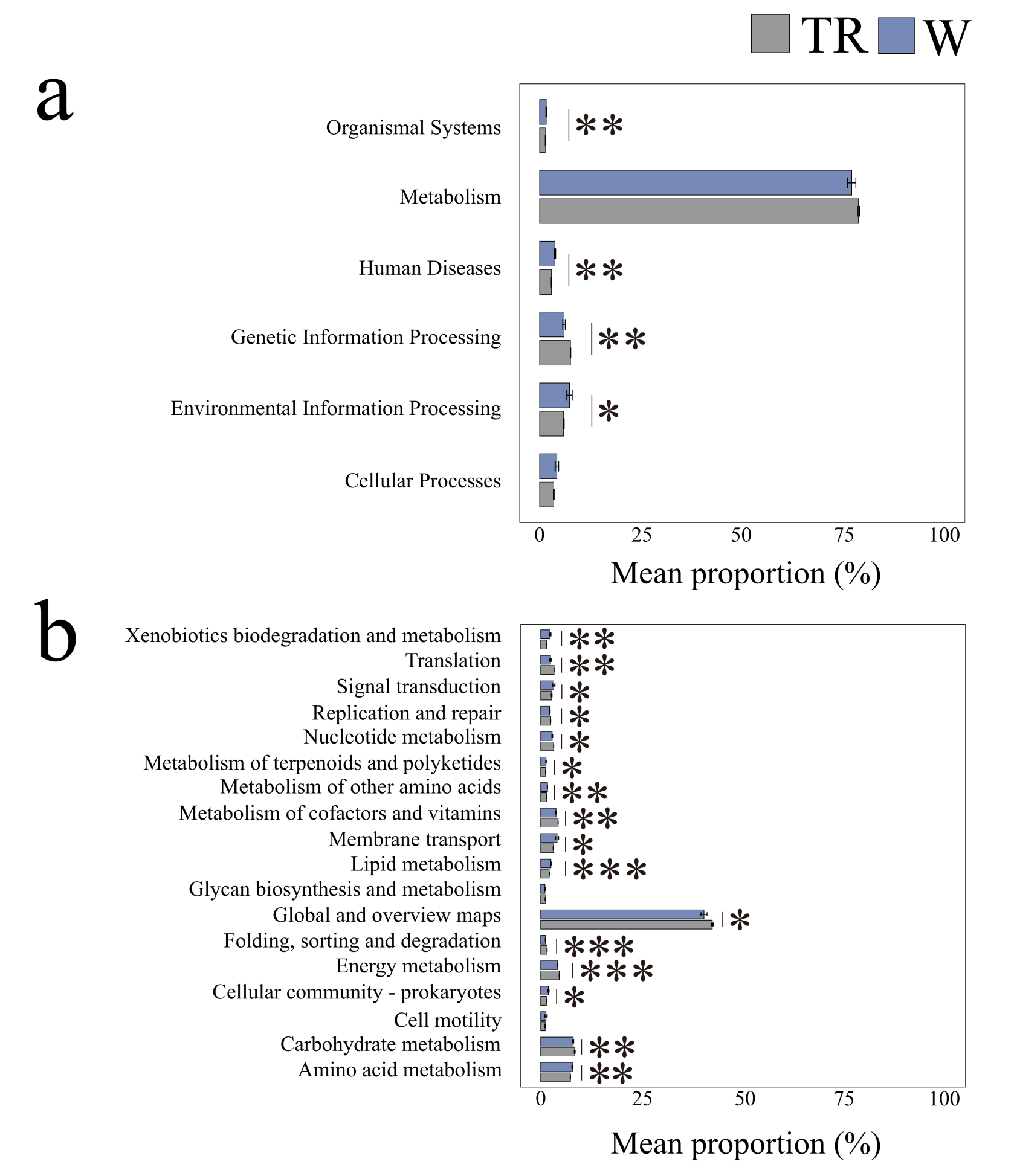

Supplement: Supplementary file 1 [file microorganisms-13-02725-s001.zip › Fig. S2.tiff]

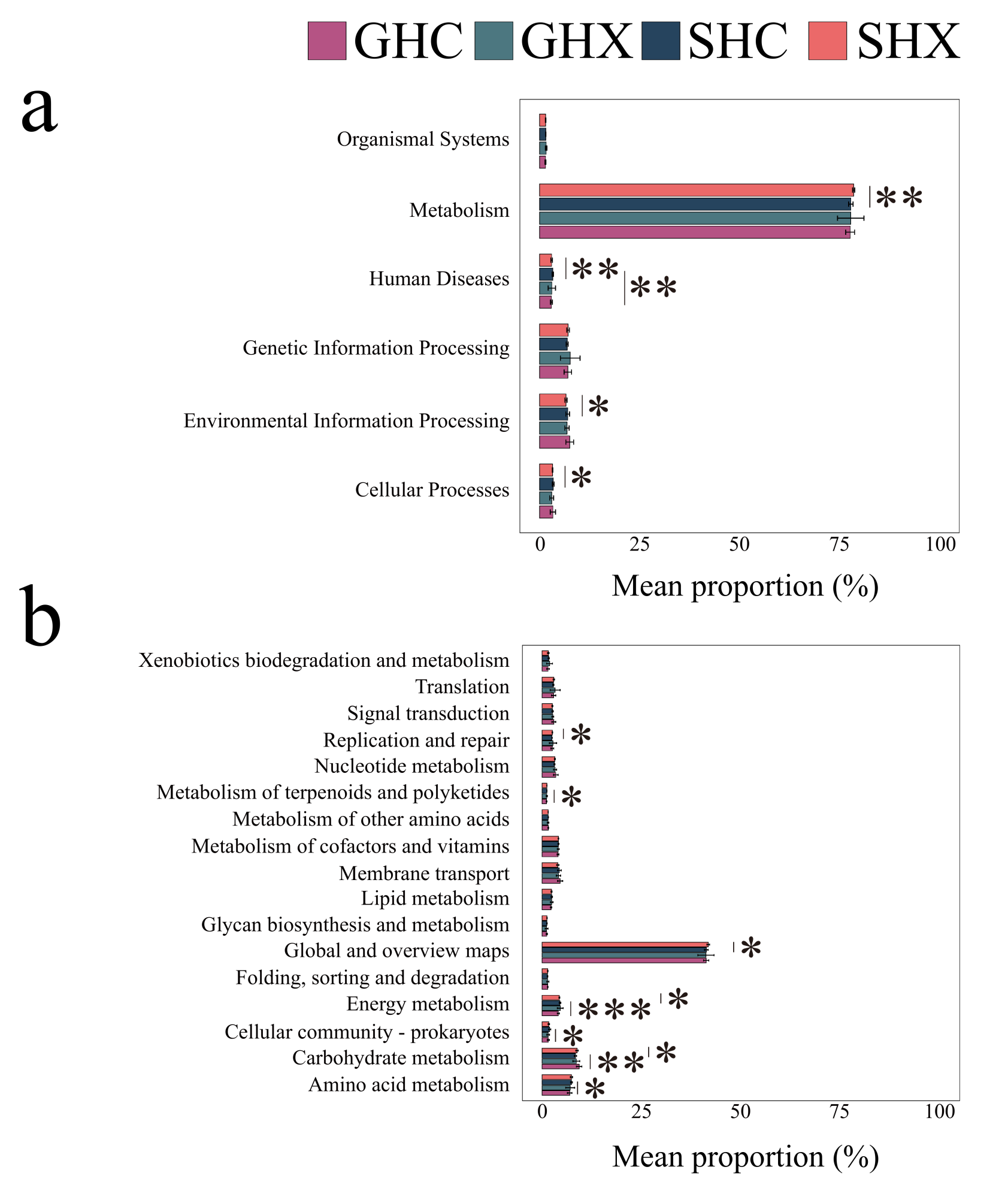

Supplement: Supplementary file 1 [file microorganisms-13-02725-s001.zip › Fig. S3.tiff]
